# What Do We Know about Diet and Markers of Cardiovascular Health in Children: A Review

**DOI:** 10.3390/ijerph16040548

**Published:** 2019-02-14

**Authors:** Pouya Saeedi, Amin Shavandi, Paula M.L. Skidmore

**Affiliations:** 1 Department of Human Nutrition, University of Otago, Dunedin 9054, New Zealand; paula.skidmore@otago.ac.nz; 2 BioMatter Unit-Biomass Transformation Lab (BTL), École interfacultaire de Bioingénieurs (EIB), Université Libre de Bruxelles, Avenue F.D. Roosevelt, 50-CP 165/61, 1050 Brussels, Belgium; amin.shavandi@ulb.ac.be; 3 Department of Medicine, University of Otago, Christchurch 8140, New Zealand

**Keywords:** cardiovascular health, diet, children

## Abstract

Chronic diseases such as cancer, diabetes, and cardiovascular diseases (CVD) are the main health concerns in the 21st century, with CVD as the number one cause of mortality worldwide. Although CVD hard endpoints such as stroke or heart attack do not usually occur in children, evidence shows that the manifestation of CVD risk factors begins in childhood, preceding clinical complications of CVD in adulthood. Dietary intake is a modifiable risk factor that has been shown to make a substantial contribution to the risk of CVD in adulthood. However, less is known about the association between dietary intake and markers of cardiovascular health in children. This review summarises the current evidence on the relationship between dietary intake and markers of cardiovascular health including traditional CVD risk factors, physical fitness, and indices of arterial stiffness and wave reflection in children. Original research published in English, between January 2008 and December 2018 fulfilling the objective of this review were screened and included. Findings show that adaptation of a healthy lifestyle early in life can be beneficial for reducing the risk of CVD later in life. Furthermore, keeping arterial stiffness low from a young age could be a potential CVD prevention strategy. However, limited studies are available on diet-arterial stiffness relationship in children, and future research is required to better understand this association to aid the development and implementation of evidence-based strategies for preventing CVD-related complications later in life.

## 1. Introduction

Healthy nutrition is one of the major parameters associated with children’s growth and cognitive development [1]. On the one hand poor or unhealthy nutrition can negatively affect children’s health, growth and cognitive performance. On the other hand over nutrition in children can also increase the incidence of various complications such as cardiovascular diseases (CVDs) risk factors and therefore resulting in poor quality of life from a young age [2].

CVDs are an ubiquitous cause of morbidity and a leading contributor to mortality worldwide, which used to be adulthood disease. However, traditional CVD risk factors such as obesity and non-alcoholic fatty liver have increased in children over the past few decades [3,4]. Increasing evidence, particularly for more novel risk factors such as interleukin-6 (IL-6), C-reactive protein (CRP), and homocysteine has also shown the underlying origins of CVD development in childhood, which persist, and indeed progress into adulthood [5]. Thus, there has been a growing interest in investigating markers of cardiovascular health and their determinants at early stages of life.

Biological, environmental, and behavioural risk factors contribute to the development and progression of the complications associated with CVD [6], of which, poor dietary intake (e.g., inadequate fruit and vegetables consumption) is a modifiable risk factor that has been shown to make a large contribution to the risk of death from CVD in adults [7]. Considering the important role of dietary intake in health, adoption of a healthy diet at young ages as an early prevention strategy could be an important step in reducing CVD risk factors and their complications from early stages of life. 

Examining individual nutrients and/or foods in relationship with health status helps to understand potential biological mechanisms responsible for the observed associations [8]. However, this approach is limited as a single food approach might not account for the synergistic or inhibitory effects of multiple nutrients in foods, whereas, dietary patterns as a more holistic approach measures foods and beverages consumed in combination [9]. Thus the investigation of dietary patterns, as a more global approach, may be complementary to traditional approaches (i.e., single nutrients/foods) and may better reflect complex relationships between food components [10,11]. Empirically derived dietary patterns using principal component analysis (PCA) in particular, is one of the most commonly used approaches in nutritional epidemiology to derive population-specific dietary patterns [12]. PCA identifies dietary patterns on the basis of the correlation between food items/groups and the degree to which they are commonly consumed together [12]. The data-dependent nature of the PCA-derived dietary patterns that takes into account foods consumed within the population of interest make them a worthy approach for assessing diet-disease relationships. 

In this review, we will provide an overview of the available literature on dietary intake, in particular dietary patterns in relationship with markers of cardiovascular health including traditional CVD risk factors (e.g., adiposity and insulin resistance), physical fitness (cardiorespiratory and muscular fitness), and indices of arterial stiffness and wave reflection, in children. This information could help health professionals to provide evidence-based nutrition prevention strategies to help children stay healthy and grow up as healthy adults.

## 2. Materials and Methods

PubMed was used as the primary search engine for our literature review to identify relevant studies on the relationship between dietary patterns and markers of cardiovascular health in children and adolescents. Search terms included dietary pattern(s), principal component analysis, PCA, arterial stiffness, wave reflection, pulse wave velocity, augmentation index, cardiorespiratory fitness, maximal oxygen consumption, maximal oxygen uptake, muscular fitness, muscular strength, fitness, cardiovascular diseases risk factors, children, and adolescents. 

All original research articles published in English, between January 2008 and December 2018 with their main objective to study the association between dietary habits and markers of cardiovascular health in children and adolescents were screened. A total of 637 studies were identified, of which 27 studies met the inclusion criteria and were used in this review. Studies were included regardless of the health status of their sample population (i.e., healthy or high-risk population of children and adolescents). Studies with their focus on the relationship between specific nutrients (e.g., vitamins and minerals) and markers of CVD were excluded. 

## 3. Dietary Patterns and Traditional CVD Risk Factors

Evidence has increasingly shown that traditional CVD risk factors such as obesity, diabetes mellitus, elevated blood pressure, and dyslipidaemia have their origin in childhood. Based on the available evidence traditional CVD risk factors in children can track into adulthood, contributing to the risk of CVD years later [13,14,15,16]. Studies have investigated factors associated with traditional CVD risk factors at this early stage of life, of which, dietary intake an important modifiable risk factor has gain lots of interest that have been explained in Section 3.1 and Section 3.2.

### 3.1. Healthy Dietary Patterns and Traditional CVD Risk Factors in Children

Research has reported an inverse relationship between healthy dietary patterns and adiposity (body mass index (BMI), percentage body fat, skinfold measurements, and fat mass) in Indian children (mean age ± SD: 9.4 ± 0.4 years old) [17], 7–11 year-old Iranian children [18], 9–11 year-old British children [19], and 11–17 year-old Brazilians [20] (Table 1). Leafy greens, fruits, cereal- and legume-based snacks, nuts, homemade sweets, breads, oily rice, meat dishes, poultry, fish, and dairy products were the main food items highly positively loaded within the healthy dietary patterns. It has been argued that healthy dietary patterns are rich in nutrients such as vitamins, minerals, and fibre, and they might be a reflection of healthy habits in general with potential benefits with regards to CVD risk factors such as obesity [21,22]. However, inconsistencies exist, as Shang et al. [23] did not find significant relationship between a ‘Healthy’ dietary pattern (positively loaded for vegetables; low-fat dairy products; legume, nuts, seeds; whole grains; and negatively loaded for high-fat dairy products; sweetened beverages; and fried chicken) and adiposity (as measured by BMI, waist circumference, percentage body fat mass) in 8–10 year-old Canadian children. 

### 3.2. Dietary Patterns with Less Healthy Foods and Traditional CVD Risk Factors 

Research in a 6–13 year-old Chinese population has shown that children with high scores for a ‘Western’ dietary pattern had significantly higher levels of low-density lipoprotein cholesterol (LDL-cholesterol), triglycerides, systolic blood pressure, and fasting glucose than children with the healthy dietary pattern. In addition, Mexican children and adolescents in the highest quintile of the ‘Western’ pattern (highly loaded for carbohydrate-containing foods such as soft drinks, snacks, and corn tortillas) had greater odds of insulin resistance than those in the lowest quintile [24]. Similarly, 9–13 year-old Greeks in the third tertile of the ‘Western’ pattern (highly loaded for margarine, sweets and savoury snacks (e.g., candies, lollipops, jellies, chips, cheese puffs and popcorn) had a greater risk of insulin resistance than those in the first tertile [25]. These findings support the hypothesis that energy-dense diets for example those high in carbohydrate/sugar may increase insulin resistance in children and adolescents and therefore causing further complications. 

Significant positive relationships have been also reported between body weight status and ‘Fast food’ pattern in 8–10 year-old Canadian children [23] and a ‘Snacking’ pattern in 5–12 year-old Colombians [26]. Surprisingly, studies in a 3–16 year-old Scottish population [27], 5–10 year-old Portuguese children [28], and 9–10 year-old Norwegian children [29] found that snacking patterns were inversely associated with overweight or obesity. The main food items in the ‘Snacks’ patterns in these studies were sweets, white bread/rolls [27], crackers/cookies and pastries [28], and foods high in fat and sugar such as French fries and cakes [29]. The inverse relationship between these dietary patterns and weight status could be, at least in part, that obese children might under-report energy-dense foods such as cookies and pastries or consumption of these types of foods might have been already restricted in their diet because of their weight status.

Overall, studies have shown that healthy dietary patterns are favourably associated with traditional CVD risk factors in childhood, whereas dietary patterns including less healthy food options are adversely associated with these risk factors. In addition to the traditional risk factors, other markers of cardiovascular health including components of physical fitness and indices of arterial stiffness and wave reflection have increasingly become the field of interest in the paediatric population. The following sections discuss the importance of these markers of cardiovascular health and their relationship with dietary intake in the paediatric population.

## 4. Physical Fitness 

When examining physical fitness, the functional status of many body systems such as the skeletomuscular, cardiorespiratory, hematocirculatory and psychoneurological system can be studied. Components of physical fitness such as cardiorespiratory fitness and muscular strength are shown to have an important role in reducing and even preventing CVD and underlying risk factors [30,31,32,33]. 

Habits formed in childhood may have a lifelong effect on health status later in life [34]. In light of evidence from longitudinal studies, cardiorespiratory fitness in children and adolescents has been a strong predictor of CVD risk factors such as adiposity, high blood pressure, and adverse lipid profile later in life [13,35,36,37,38,39,40,41]. Findings from cross-sectional studies in children and adolescents have also shown that high levels of cardiorespiratory fitness are significantly inversely associated with traditional and emerging CVD risk factors such as adiposity, clustering of metabolic risk factors, and homocysteine levels [42,43,44,45,46,47,48,49], while positively associated with bone mass and high density lipoprotein cholesterol (HDL-cholesterol) [48,49]. As evidence shows cardiorespiratory fitness is beneficially associated not only with children’s current health status, but also when they grow older. 

Muscular strength, another important health-related component of the physical fitness is an important marker of health in children and adolescents. Studies have reported an inverse relationship between muscular strength with both traditional and emerging CVD risk factors such as insulin resistance, systolic blood pressure, lipid-metabolic index (triglyceride, low density lipoprotein cholesterol; LDL-cholesterol, HDL-cholesterol, and glucose concentrations), clustered metabolic risk (sum of z-scores of waist circumference, systolic blood pressure, triglycerides, total cholesterol/HDL-cholesterol ratio, and insulin resistance), and C-reactive protein, complement component 3 (C3), and ceruloplasmin [39,50,51].

As mentioned in Section 1, a healthy diet as a component of an overall healthy lifestyle is favourably associated with traditional CVD risk factors in childhood. High levels of cardiorespiratory fitness and muscular strength are also beneficially associated with CVD risk factors in children. It is therefore important to understand the interaction between dietary intake and cardiorespiratory fitness and muscular strength in children. This may lead to designing intervention and prevention strategies targeting modifiable risk factors. These strategies can help to promote positive behaviours and reduce undesirable health outcomes in childhood and consequently to reduce/prevent the development of CVD in adulthood. 

### 4.1. Relationships between Diet and Cardiorespiratory Fitness

Studies in children have assessed the relationship between diet mainly eating habits, and cardiorespiratory fitness [52,53,54,55,56,57] (Table 2), of which, one study found that unhealthy eating habits (e.g., uncontrolled or restrained eating) were associated with higher levels of relative VO_2peak_ (an indicator of cardiorespiratory fitness) in an 8-16 year-old Spanish cohort [55]. Unlike Martín-García and colleagues, other studies have reported that healthy eating habits (i.e., regular breakfast consumption) are positively associated with cardiorespiratory fitness in children and adolescents [52,53,54,56,57], which could be attributed in part to having a healthier lifestyle in general.

Three studies have examined the associations between dairy consumption and cardiorespiratory fitness [58,59,60]. A significant positive association has been reported between dairy consumption and cardiorespiratory fitness in 9–13 year-old Greeks, 12–17 year-old Europeans, and 14–19 year-old Brazilians [58,59,60]. Dairy products are rich sources of essential amino acids, vitamins (e.g., B2 and B12), and minerals (e.g., zinc and calcium) [61,62]. The essential amino acids content of dairy products plays an important role in the synthesis of muscle protein [63]. Vitamin B2 and B12 have recognized roles in energy production, by improving oxygen transport to tissues and the oxidisation of fatty acids, which may partly explain the favorable relationship between dairy consumption and cardiorespiratory fitness [64,65]. Evidence from cross-sectional and follow-up studies has shown a beneficial association between milk consumption and adiposity in children, which may be attributed to calcium content of milk and its role in fat metabolisation and oxidation [66]. Since, adiposity is closely and inversely associated with performance in cardiorespiratory fitness tests, this may explain the favorable relationship between milk consumption and cardiorespiratory fitness [67]. Furthermore, dairy consumption in children may be a representative of a good quality diet in general [68]. This can also partly explain the favourable relationship between milk consumption and cardiorespiratory fitness.

A relatively small body of literature has examined the relationship between dietary patterns and cardiorespiratory fitness [69,70,71]. Howe et al. [70] found a positive association between the ‘Fruits and Vegetables’ pattern (highly positively loaded for fruit, vegetables, cheese and brown/multigrain bread) and cardiorespiratory fitness in 14–18 year-old New Zealanders. Similarly Grao-Cruces et al. [69] reported that adherence to the Mediterranean dietary pattern (e.g., high consumption of fruits, vegetables, olive oil, cereals, legumes, and nuts) was positively associated with cardiorespiratory fitness ($\dot{V}$O_2max_) in 12–16 year-old Spanish adolescents. It can be suggested that healthy eating patterns and higher levels of cardiorespiratory fitness are reflections of a healthy lifestyle in general. Furthermore, healthy dietary patterns such as the Mediterranean diet and a pattern highly loaded for fruits and vegetables may provide the body with high amounts of antioxidants that reduce oxidative stress and the production of reactive oxygen species [72]. The antioxidant activity of foods such as fruits, vegetables, nuts, and fish can reduce damage from oxidative stress, which may explain, in part, the beneficial relationship between healthy dietary patterns and cardiorespiratory fitness.

In addition, Howe et al. [70] found a negative association between a ‘Treat Foods’ pattern (i.e., sweets, chocolate confectionery, potato crisps, sugar-sweetened and artificially sweetened soft drinks) and cardiorespiratory fitness in New Zealand adolescents [70]. Also, consumption of sweetened beverages (i.e., juices, carbonated, soft and isotonic drinks) was negatively associated with cardiorespiratory fitness in European girls [58]. Foods highly loaded in the less healthy dietary patterns such as potato crisps, sugar-sweetened beverages, and sweets are considered as energy-dense foods with poor nutritional value. They are considered unhealthy, with an adverse effect on health outcomes such as cardiovascular and metabolic health [73]. Consumption of these types of foods are significantly associated with weight gain in children [74]. Adiposity limits performance in weight bearing activities and this could be a possible reason for observing the negative association between less healthy dietary patterns and cardiorespiratory fitness.

However, a recently published study in 9–11 year-old healthy population of children did not find a meaningful relationship between either “Snacks” (highly positively loaded for salty/sweet snacks, sweetened beverages, ice cream, white bread, and pasta) or “Fruit and Vegetables” (highly positively loaded for fruit, vegetables, dairy products, and meat) patterns and cardiorespiratory fitness. The authors have suggested that in a cohort of apparently healthy children who were mainly (76%) normal weight, there was no association between food choice and cardiorespiratory fitness [71]. 

So far, only three studies, one in Spain and two in New Zealand, have examined the relationship between dietary patterns and cardiorespiratory fitness in children and adolescents. Different dietary assessment methods have been used to identify dietary patterns in these studies: adherence to the Mediterranean diet [69] and PCA-derived dietary patterns [70,71]. Two of the studies were in adolescents (12–18 years) and one in children (9–11 years). Adolescents become more independent from their parents with less parental control over their diet than younger children. Therefore, younger children may have different eating habits and consequently different dietary patterns than adolescents. 

### 4.2. Relationships between Diet and Muscular Strength 

There is a scarcity of research on the relationship between dietary intake and muscular strength in children (Table 2). Two studies have examined the relationship between dietary habits (breakfast consumption) and muscular strength in children and adolescents [52,75], where there was no significant association between breakfast consumption and muscular fitness (handgrip strength) in 6–11 and 12–15 year-old Europeans [52,75].

In addition, four studies have determined the association between individual foods and muscular strength [60,75,76,77]. No significant associations were found between daily milk consumption (β = −0.02 kg, *p* = 0.782) [60], frequency of fruit and vegetable intake (β = 0.012 kg, *p* = 0.155) [75], and amount of daily fruit and vegetable consumption [76] with handgrip strength in 9–13 year-old Greeks, 6–11 year-old Europeans, and 5–18 year-old Americans, respectively. Only a study by Neville et al. [77] found that Irish girls (12 and 15 years) in the lowest tertile of fruit and vegetable consumption had significantly lower handgrip strength than those in the highest tertile of fruit and vegetable consumption (β = −0.75 kg, 95% CI = −1.35, −0.15 kg). The beneficial effect of fruit and vegetable consumption on muscular strength has been partially attributed to the antioxidant activity of the biologically active nutrients such as carotenoids and vitamin C in fruits and vegetables [78,79]. Antioxidants play an important role in protecting various cellular components such as cell membranes and proteins from the catabolic effects of oxidative stress [80,81]. Oxidative stress is the imbalance between the production of reactive oxygen species and antioxidant defence [82]. High levels of reactive oxygen species may change membrane function and therefore tissue permeability by inducing lipid peroxidation and inactivating the membrane receptors and enzymes [83]. Reactive oxygen species can also increase susceptibility to proteolysis by breaking the peptide chains, inducing amino acids oxidation, and cross-linking of proteins [84]. These cellular changes may therefore impair the normal performance of muscles. 

Despite findings by Neville and colleagues, it should be noted that humans eat food with a wide range of nutrients with possible synergistic and/or interactive relationships. Thus, analysing the association between specific aspects of diet and muscular strength may not provide sufficient information. Two studies, one in a 12–16 year-old Spanish population and the other one in 9–11 years old New Zealand children are the only studies that have examined the relationship between dietary patterns and muscular fitness [69,71]. Grao-Cruces and colleagues [69] did not find a significant relationship between adherence to the Mediterranean diet (a priori dietary pattern approach) and handgrip strength in Spanish adolescents. Similarly, Saeedi et al. [71] did not fond a meaningful relationship between a posteriori derived dietary patterns and muscular fitness in New Zealand children. The results suggest that other factors than diet might play a more important role with regards to muscular fitness during early stages of life.

## 5. Arterial Stiffness

Arterial stiffness, that is the rigidity of the arterial wall, is an early event in the development of cardiovascular damage and is considered an independent predictor of cardiovascular morbidity and mortality [85,86,87]. Globally, CVD accounts for millions of deaths each year [88]. As arterial stiffness advances, the function of the aorta (i.e., transferring nutrient-rich blood to the peripheral arteries and body organs) will be compromised [89]. Stiff arteries put stress on body organs such as the heart, brain, and kidneys and therefore increases the risk of CVD and all-cause morbidity and mortality [89,90,91,92,93,94]. Although CVD hard endpoints such as myocardial infarction, stroke, and heart failure do not usually exist in the paediatric population, evidence increasingly shows that the arterial stiffening process begins in childhood [95,96,97]. Indeed, changes in arteries (structural and functional) may be identifiable from childhood [95,96,98]. 

Pulse wave velocity (PWV) and augmentation index (AIx) as independent predictors of CVD provide useful information about arterial stiffness and wave reflection, respectively. However, these two measures cannot be used interchangeably. PWV is the gold standard measure of arterial stiffness and one of the most powerful measures to predict future CVD [92]. AIx measures the impact of the reflected waveforms from periphery on measures of central hemodynamics [99]. There is an increasing interest on the assessment of measures of central hemodynamics than the peripheral blood pressure. Central hemodynamics are more sensitive to pharmacological therapeutic interventions and have increased value for cardiovascular risk assessments than the peripheral blood pressure [100]. Overall, AIx increases in parallel with PWV throughout life, but reaches a plateau at about the age of 60 years. Although AIx and PWV are associated, they determine different information about cardiovascular health that are complementary [101].

Therapeutic strategies such as pharmacological treatments and changing unhealthy behaviours such as smoking and low levels of physical activity have shown beneficial effects on arterial stiffness and consequently CVD-related complications in adults [102,103,104]. Randomised controlled trials (RCTs) in adults have highlighted the importance of dietary intake on arterial health and found that dietary patterns such as the Dietary Approaches to Stop Hypertension (DASH) diet and consumption of foods such fermented milk products reduce arterial stiffness [105,106,107,108]. Studies in adults have provided useful information on the favourable effect of modifiable factors such as the dietary intake on arterial stiffness [105,106,107,108,109]. The application of therapeutic strategies in adults may impose a large burden from both the public health and financial perspectives. As changes in arteries start from the first decade of life [95,96,97], keeping the arterial stiffness low from a young age rather than applying therapeutic strategies years later when high levels of stiffness have been already developed may be a better strategy. Therefore, understanding diet-arterial stiffness relationship in early stages of life may help to devise intervention strategies to slow down the arterial stiffening process, as children grow older.

### Relationship between Single Nutrients and/or Food Items and Indices of Arterial Stiffness and Wave Reflection

A limited number of studies in the paediatric population have examined the relationship between dietary intake and indices of arterial stiffness and/or wave reflection (Table 3). Of which, two studies have measured the association between single nutrients and/or food items and indices of arterial stiffness in 12–15 year-old overweight/obese adolescents [110] and 10 year-old healthy-weight children [111]. 

Arnberg and colleagues [110] assessed the independent relationships between milk consumption and percentage energy from protein with AIx@HR75 and PWV in 12–15 year-old overweight Danish adolescents. They did not find significant associations between milk consumption and percentage energy from protein (4-d self-reported precoded food record) with AIx@HR75. In the multivariate analysis, pedometer counts were the only significant predictor of AIx, adjusted for age, gender, Tanner stage, mean BP, heart rate, height, BMI, and physical activity. After adjusting for biochemical measures (i.e., HDL-cholesterol, triglyceride, and HOMA), the significant relationship between pedometer counts and AIx was attenuated, and HOMA was found to be the only significant predictor of AIx. The coexistence of several CVD risk factors in obese populations may affect the endothelial function and consequently measures of wave reflection such as AIx [112,113]. This is also in accordance with the fact that obese children and those with obesity-related type 2 diabetes are at higher risk of having stiffer arteries than non-obese children [114,115]. A large number of participants (90%) in Arnberg et al. [110] study were overweight and obese, which may explain why AIx@HR75 was only significantly associated with HOMA. 

Arnberg and colleagues [111] found a positive association between percentage energy from protein and PWV. Usually it is expected that protein intake, which is a substrate for nitric oxide (NO) production, will have a favourable effect on arterial stiffness since NO may regulate stiffness of large arteries [116,117]. Total protein intake was positively correlated with serum urea concentration and they have, to a large extent, attributed the unfavourable association of protein and PWV to meat consumption. Total dietary protein intake represents consumption of a variety of food items (e.g., red meat, fish, poultry, legumes, nuts, etc.), with divergent health effects [118]. It would have been useful if authors had provided information on the types of meat and other sources of dietary protein intake such as legumes. Assessing the impact of nutrient intake (total protein intake) per se on cardiovascular health seems to be less informative than examining the food intake. In agreement with this, a recent meta-analysis of RCTs by Schwingshackl & Hoffmann [119] found neither beneficial nor unfavourable effects of protein intake on CVD risk factors (e.g., waist circumference, LDL-cholesterol, HDL-cholesterol, systolic BP, diastolic BP, inflammatory markers such as C-reactive protein, HbA1c, etc.).

In addition, Arnberg et al. [110] found an inverse association between milk consumption and cf-PWV. They have argued that this relationship is based on the calcium content of milk and its effect on calcitriol suppression and decreasing calcium in smooth muscle cells, and therefore a reduction in peripheral resistance [120]. A recent systematic review of RCTs in adults examining the effect of cholecalciferol supplementation (precursor of calcitriol) on arterial stiffness has reported no significant effect [121]. Other factors may have been involved in the beneficial relationship between milk and arterial stiffness. Milk consumption in children could be a representative of a high quality diet in general [68] and consequently healthier cardiovascular risk profile. 

In another study [111] it is shown that total fat intake is positively associated with PWV in 10 year-old Danish children. High fat consumption can be a reflection of a poor quality diet, which may be also high in sugar and refined grains, and low in foods such as fruit, vegetables, and legumes. Evidence has shown that there is an adverse relationship between such a diet and health complications such as obesity, diabetes, and hypertension [122], which are unfavourably associated with arterial stiffness progression [123,124].

Relationship between dietary patterns and indices of arterial stiffness and wave reflection.

Only two studies have assessed the association between dietary patterns and indices of arterial stiffness and wave reflection, where both studies were in high-risk population of children and adolescents: 10-19 year-old diabetic children and adolescents [125] and 12 year-old overweight/obese adolescents [126]. Lamichhane et al. [125] examined the relationship between a dietary pattern highly positively loaded for sweetened beverages, diet soda, eggs, and high-fat meats with indices of arterial stiffness and wave reflection i.e., in the study by Lamichhane and colleagues. Although there was an adverse significant relationship between the dietary pattern and PWV in the bivariable model, after adjusting for potential confounders the association was attenuated. The estimated effect size in the multivariate model was small (0.01 m/s) and suggested no significant relationship between the dietary pattern and cf-PWV (mean PWV: 5.39 ± 0.84 m/s). 

Lydakis et al. [126] determined the association between the degree of adherence to the Mediterranean diet (KIDMED index) with AIx and reflected wave transit time/height (RWTT/H) in 12 year-old Greeks. The RWTT/H has an inverse relation with PWV and is used as an index of brachial arterial stiffness. There was a significant inverse association between the KIDMED index and AIx. The high prevalence of overweight and obesity (43.3%) in their sample population and the adverse relationship between the KIDMED index (an indicator of healthy eating habits) and AIx may suggest the relationship between healthy lifestyle and a healthy heart, although they did not find a significant relationship between the KIDMED index and RWTT/H. However, findings from this study are difficult to interpret appropriately, as inconsistencies exist in the reported methodology.

Overall, two studies have examined the relationship between dietary patterns and indices of arterial stiffness/wave reflection and both studies were in high-risk populations (i.e., participants with type 1 diabetes and high prevalence of overweight and obesity) [125,126]. The other two studies examined the relationship between a nutrient and/or food item intake with indices of arterial stiffness, of which one was in overweight and obese adolescents and the other one in healthy children [110,111]. Findings from these studies provide some support for the importance of a healthy dietary intake on vascular health at early stages of life. Studies in children are, however, limited and comparing their findings is difficult due to their sample population (healthy vs. diseased populations: diabetic or overweight), assessment of different dietary factors (nutrient intake vs. dietary patterns), and the use of various indices of arterial stiffness (PWV vs. RWTT/H and AIx vs. AIx@HR75). Further research is required to better understand the association between dietary intake and arterial stiffness in children, which will aid the development and implementation of evidence-based strategies for preventing CVD-related complications later in life.

## 6. Conclusions 

Nutrient deficiency-related complications were the main health concerns in the early 20th century, whereas nowadays, poor quality of diet and food choices are of concern [127]. Eating behaviours are formed during childhood, a period when optimal nutrition is important for the maintenance of the growth and ensuring a healthy future [128,129].

This review provided a summary of the available research on the importance of dietary intake in relationship with markers of cardiovascular health in the paediatric population. Both healthy dietary patterns and physical fitness are shown to be beneficially associated with children’s health [19,20,31,130]. The relationships between dietary patterns and specified components of physical fitness have provided a reflection of children’s lifestyle in general, with potential public health benefits. Adaptation of a healthy lifestyle early in life can be beneficial for reducing the risk of occurrence of CVD later in life.

In addition, arterial stiffness is a strong predictor of CVD- and all-cause mortality in the high-risk and general adult populations [95,131,132]. In adults the important role of healthy dietary intake on arterial health have been highlighted [105,106,107,108]. Considering that changes in arteries start from a young age, during the first decade of life [95,96,97], keeping the arterial stiffness low from a young age could be a potential CVD prevention strategy. There is very limited studies on diet-arterial health relationship in children and future research is required to better understand this association, which will aid the development and implementation of evidence-based strategies for preventing CVD-related complications later in life. This information can help healthcare professionals, paediatricians, nutritionists, and researchers working in the relevant fields to provide evidence-based nutrition strategies to help children stay healthy and grow as healthy adults.

## Figures and Tables

**Table 1:** PCA derived dietary patterns and their association with traditional CVD risk factors in children.

Reference	Subjects	Dietary Intake Assessment Method	Derived Patterns (Explained Variance %)	Health Outcome	Results
[17]	9.4 ± 0.4 y South India	FFQ	1. ‘Snack and Fruit’ 2. ‘Lacto- vegetarian’	Adiposity	Positive association: Snack and Fruit with BMI, subscapular skinfold & percentage body fat No association: Lacto-vegetarian pattern with BMI, skinfolds, percentage body fat
[20]	11–17 y Brazil	FFQ for adolescents	1. ‘Junk food’ 2. ‘Healthy’ 3. ‘Traditional’	BMI	Positive association: Healthy pattern and overweight
[23]	8–10 y Canada	Three non-consecutive 24-h recalls	1. ‘Traditional’ 2. ‘Healthy’ 3. ‘Fast food’	Adiposity	The ‘Traditional’ and ‘Healthy’ patterns were not associated with adiposity. Children in the 75th percentile of ‘Fast food’ pattern were more likely to be overweight than those in percentiles <25th. Also they had higher fat mass percentage and waist circumference.
[18]	7–11 y Iran	FFQ	1. ‘Healthy’ 2. ‘Western’ 3.‘Sweet and dairy’	BMI	Boys: No significant associations Girls: Children in the second quartile of ‘Healthy’ dietary pattern were more likely to have higher BMI than those in the highest quartile of ‘Healthy’ pattern. Those in the second quartile of ‘Western’ pattern had significantly lower BMI than those in the forth quartile. Children who had lower adherence to ‘Sweet and dairy’; dietary pattern had lower BMI
[27]	3–16 y Scotland (results for 5–11 y are presented)	FFQ	Boys: 1. ‘Fruit and vegetables’ 2. ‘Snacks’ 3. ‘Fish & sauce’ Girls: 1. ‘Fruit and vegetables’ 2. ‘Puddings’ 3. ‘Snacks’	BMI	In boys only ‘Snacks’ pattern was positively associated with BMI (*p* trend= 0.047) ‘Fish & sauce’ pattern was negatively associated with BMI (*p* trend= 0.023)
[29]	9–10 y Norway	FFQ	1. ‘Snacking’ 2. ‘Junk/convenient’ 3. ‘Varied Norwegian’ 4. ‘Dieting’	BMI	Tertiles of dietary patterns: Lower tertile of ‘Junk/convenient’ & upper tertile of ‘Dieting’ were negatively associated with highest incidence of overweight Upper tertiles of the ‘Varied Norwegian’ was negatively associated with overweight
[28]	5–10 y Portugal	Semi quantitative FFQ	Eight dietary patterns were identified	BMI	‘Pattern 6’ was negatively associated with Obesity ‘Pattern 7’ was positively associated with Obesity
[24]	7–18 y Mexico	Semi-quantitative FFQ	1. Western 2. Prudent 3. High protein/fat	Insulin resistance	Higher quintile of the ‘Western’ pattern was associated with increased odds of Insulin resistance.
[25]	9–13 y Greece	Three 24 h recalls	Five dietary patterns were identified	Insulin resistance	Insulin resistance was negatively associated with ‘Pattern 3’

BMI: Body mass index, CI: confidence intervals, PCA: Principal component analysis.

**Table 2:** Dietary intake and measures of physical fitness.

[Reference] Study Sample	Aim	Dietary Intake	Cardiorespiratory Fitness	Results	Muscular Strength	Results
Food intake: Nutrients, food items, dietary pattern						
[75] Based on IDEFICS data 4903 Europeans 6–11 y	To examine determinants of physical fitness	Non-quantitative FFQ	20 msrt	Physical fitness was negatively associated with age, BMI z-score, parental education, mother’s BMI, while positively with sex (girls as ref), psychosocial well-being, physical activity	Handgrip strength: Takei Bipedal position with arm extended Score: average of right & left	No significant association between fruit & vegetable/breakfast consumption with handgrip strength.
[60] Healthy Growth Study 600 Greek children 9–13 y	To determine the associations of milk consumption with fitness, anthropometric and biochemical indices	3-d 24-h recalls	20 msrt	Milk consumption (mL/d) was positively associated with number of stages in 20 msrt	Handgrip strength	No association
[69] 1988 Spanish adolescents 12–16 y	To examine the association between aerobic, musculo-skeletal, and motor capacity and life satisfaction, risk behaviour, and adherence to the Mediterranean diet in adolescents	KIDMED (adherence to Mediterranean diet	20 msrt, Leger’s protocol $\dot{V}$O_2max_, Leger’s equation FITNESSGRAM standards: low & high	Positively: life satisfaction, adherence to Mediterranean diet (β = 0.069, *p* = 0.019)	Handgrip strength: Takei Standing position with arm straight Score: Twice and sum of best results in each hand Category: above/below the mean: low and high	Boys: 61 ± 16.7 kg and girls: 46.5 ± 9.47 kg Positively: alcohol use. Drunkenness No association with adherence to Mediterranean diet (β = 0.019, *p* = 0.539)
[76] 82 native American youth 5–18 years	To determine correlation among physical fitness, dietary intakes, activity levels, and BMI.	Energy and macronutrient intakes: one 24-h recall	President’s Physical fitness Awards program	No significant associations were found	President’s Physical fitness Awards program: partial curl-ups (abdominal strength), and right-angle push-ups	No significant associations were found
[77] Young Hearts 2000 study 2017 Irish adolescents 12 and 15 years	To determine relationships between fruit and vegetable consumption and muscle strength and power.	7-d diet history One-to-one interview, habitual intake, especially fruits and vegetables	-	-	Handgrip strength: Takei Standing position with arm straight Two measurements for each arm The highest of measurements to be used in analyses	Boys: 26.9 ± 9.06 kg Girls: 21.5 ± 5.08 kg Girls: significant relationship between fruit and vegetable consumption and handgrip strength in Models 1,2,3
[58] Based on HELENA data 1492 Europeans (8 countries) 12–17 years	To determine relationships between cardiorespiratory fitness and dietary.	2-d 24-h recalls Computer-based Self-reported	20 msrt	Mean daily intake of dairy products and fruits was positively associated with CRF in boys and girls. Sweetened beverage and grain/potatoes were negatively associated with CRF in girls.	-	-
[59] Based on BGPFERHLH data 879 Brazilians 14–19 years	To analyse the association between erobic fitness levels and socio-demographic factors, lifestyle and excess body fatness	Adequate/inadequate milk consumption Adequate/inadequate soft drinks	Modified Canadian Aerobic Fitness Test $\dot{V}$O_2max_	Inadequate milk consumption was only significantly associated with cardiorespiratory fitness in girls.	-	-
[70] 279 New Zealanders 14–18 years	To determine the association between cardiorespiratory fitness and dietary patterns	Non-quantitative FFQ	20 msrt	Positive relationship between cardiorespiratory fitness and ‘Fruits and Vegetables’ pattern.	-	-
[52] HELENA study. Eight study centres in Europe 3528 participants 12.5–17.5 years	To determine the association between breakfast consumption and components of physical fitness including	Breakfast habit: ‘consumer’, ‘occasional consumer’ and ‘skipper’	20 msrt	Cardiorespiratory fitness was positively associated with breakfast consumption in boys, but not in girls	Handgrip strength	No significant associations were found
[57] 278 French children 6–10 years	To determine associations between eating habits with aerobic fitness and lower limb muscle power.	Eating habits: frequency of breakfast consumption, snack between meals, and type of drink while thirsty	20 msrt, Leger’s protocol	Children who never or sometimes ate breakfast had lower cardiorespiratory fitness than children who had breakfast everyday.	Lower limb explosive strength: Squat Jump	Children eating breakfast sometimes had lower squat jump than those eating breakfast everyday
[53] Part of HELENA study 10 European countries 2929 adolescents 12.5–17.5 years	To determine relationships between breakfast consumption and CVD risk factors.	Breakfast habits: ‘I often skip breakfast’	20 msrt, Leger’s protocol $\dot{V}$O_2max_	Breakfast consumers had higher $\dot{V}$O_2max_ than occasional consumers and skippers Breakfast consumers had higher $\dot{V}$O_2max_ than occasional consumers and skippers	-	-
[56] 4326 English participants 10–16 years	To determine relationships between habitual breakfast consumption in school days, BMI, physical activity, and cardiorespiratory fitness.	School-day breakfast habits	20-metre PACER test Performance	Compared with boys always ate breakfast, boys who never ate breakfast were more likely to have low cardiorespiratory fitness. There was no association between CRF and breakfast habits in girls.	-	-
[54] Southern California 10–12 years	Hypothesized that daily breakfast consumers would be more likely to be in the healthy fitness zone than those that consume breakfast less frequent.	Breakfast consumption frequency	20-metre PACER test	Daily breakfast eaters were 3.82 times more likely to be in the healthy fitness zone for PACER run compared with those sometimes consumed breakfast	-	-
[55] 192 children and adolescents 8.8–16.8 years	To determine the connections between eating behaviours and body composition and cardiovascular levels	Eating habits: i.e., ‘Cognitive Restraint’, ‘Uncontrolled eating’, and ‘Emotional eating’	Peak oxygen uptake	Being in low tertile of emotional eating showed lower values of relative VO2 peak in comparison to the high and medium tertile. Being in ‘low’ tertile of cognitive Restraint showed higher values of relative VO2 peak than the high tertile.	-	-

BMI: Body mass index, CI: Confidence intervals, CRF: cardiorespiratory fitness, CVD: Cardiovascular diseases, FFQ: Food frequency questionnaire, HELENA: Healthy Lifestyle in Europe by Nutrition in Adolescence-Cross-Sectional Study, BGPFERHLH: Brazilian Guide of Physical Fitness Evaluation Relatedto Health and Life Habits, IDEFICS: IDentification and prevention of dietary and lifestyle induced health EFfects In Children and infantS, PACER: Progressive aerobic cardiovascular endurance run, $\dot{V}$O_2max_: Maximal oxygen consumption, $\dot{V}$O_2peak_: Peak oxygen uptake.

**Table 3:** Studies on the relationship between dietary intake (nutrients/patterns) and indices of arterial stiffness in children and adolescents.

[Reference], Study Sample	Aim	Method	Findings
[125] United States n = 1153/1347 10–19 years Youth with type 1 diabetes	To determine associations between baseline dietary pattern/s with AIx, PWV, and brachial distensibility measured later in a cohort with T1 diabetes.	Diet: 85-item FFQ, AIx@HR75 and PWV, brachial distensibility	Dietary pattern score was significantly positively associated with AIx@HR75.
[110] Copenhagen n = 193 children Overweight children 12–15 years	To examine relationships between protein intake, milk intake, physical activity, adiposity, and arterial stiffness in overweight children with habitual milk intakes ≤250 mL/d.	Diet: 4-d self-reported precoded food record cf-PWV and AIx@HR75	There was a positive relationship between %energy from protein and cf-PWV (ß = 0.05; *p* < 0.01), whereas milk intake was negatively associated with cf-PWV (ß= −0.64, *p* = 0.05). AIx@HR75 was negatively associated with pedometer counts (ß = −3.66; *p* < 0.05), there were no significant relationship between %energy from protein and milk consumption with AIx@HR75.
[111] The Copenhagen Cohort Study on Infant Nutrition and Growth Denmark. Healthy children 10 years n = 93 boys and girls	To assess current determinants of arterials stiffness in 10 year-old children	Average daily intake of fat and energy: precoded 7-d food record, parent proxy Aorta-radial PWV and aorta-femoral PWV	%energy from fat positively related to AR-PWV (ß = 3.1, 95% CI = 0.9; 5.2) and AF-PWV (ß = 1.8, 95% CI = 0.2; 3.2). After adjusting for total energy intake, only AR-PWV was remained significant. Energy intake negatively related to radial PWV (ß = −6.4, 95% CI = −11.7; −0.8)
[126] Greece, Heraklion, Southern Healthy participants n = 287 First grade of high school (12 years)	To assess the correlation of obesity, BP, and dietary habits (adherence to the Mediterranean diet) with indices of arterial stiffness.	Diet: KIDMED index AIx, PPP/CPP, RWTT/H	Independent negative correlation between KIDMED index and AIx (ß = −0.114, *p* = 0.026). There was no significant relationship between KIDMED index and PPP/CPP and RWTT/H.

AF-PWV: aorta-femoral pulse wave velocity, AIx: Augmentation index, AIx@HR75: Augmentation index for a heart rate of 75 beats per minute, AR-PWV: Aorta-radial pulse wave velocity, ß: Beta coefficient, BMI: Body mass index, BP: Blood pressure, CI: Confidence intervals, DXA: Dual energy X-ray absorptiometry, DV: Dependent variable, FFQ: Food frequency questionnaire, IDV: Independent variable, IOTF: International obesity task force, PPP/CPP: Peripheral pulse pressure to central pulse pressure ratio, PWV: Pulse wave velocity, RWTT/H: Reflected wave transit time/height.
